# The Poisonous Action of Potassium Chlorate

**Published:** 1887-06

**Authors:** William B. Hills


THE POISONOUS ACTION OF POTASSIUM CHLORATE.

The view first advanced by Fourcroy and Alyon, that potassium chlorate, when taken into the system, gives up part of its oxygen, has, since the investigation of Binz* and Marchand,† been generally accepted in explanation of the poisonous action of the salt. B. J. Stokvis‡ contradicts this view, basing his opinion upon investigations of W. C. Kimmyser,§ of H. C. M. v. Gorcom, and of his own.

Kimmyser has investigated the elimination of the chlorate in his own urine, and in that of rabbits and dogs. He found that, of four grammes of sodium chlorate, there was eliminated unchanged, in his own urine, 2.277 grammes, and in the urine of a dog, 3.618 grammes. In the urine of rabbits, to which one and sixteen grammes had been administered, he found 0.896 gramme and 13.53 grammes, respectively. On the days when the chlorate was administered, there was an increased excretion of chlorides (and usually of urea); but this increase, according to the author, is not to be attributed to a

* Archiv. für experim. Pathol., 1879, p. 153.

† Archiv. für pathol. Anat., 1879.

‡ Berichte der deutschen chemischen Gesellschaft, 1886, p. 778, from Archiv. für experimen. Pathol., 21, p. 169.

§ Academ. Prœfschrift, Amsterdam, 1884.

reduction of the chlorate, but to a drain on the system, since, on the following days, there was a diminution in the amount of chlorides excreted, the chlorate behaves, in this respect, like sodium nitrate, as Kimmyser determined by experiments on rabbits, whose urine had been rendered free from chlorides by inanition. He, therefore, concludes that a reduction of the chlorate in the system is not proven. Such reduction is also denied by Stokvis, Isamburt, Rabuteau, and Von Mering. The small amount of chlorate unaccounted for in the urine is explained, according to Stokvis, by slow elimination, by passage into the secretions, which takes place even after the ingestion of small doses, by reduction in the urine, and by experimental errors. Kimmyser finds that, when urine containing one per cent. of sodium chlorate is allowed to stand twenty-four hours at 20° C., twenty-four per cent. of the salt is reduced This reduction does not take place at 0° C., or in acid urine and is less when the urine has been boiled. It depends, according to the author, on beginning putrefaction. Reduction in the blood depends on complicated putrefactive processes, which take place only when the blood loses its vitality. There is formed, in this case, methæmoglobin and hæmatin. An increased temperature favors the process, but a reduction of the whole quantity of chlorate present takes place only rarely, and when the amount of salt is small.

The appearance of the blood observed in fatal cases o, potassium-chlorate-poisoning is attributed, by the author, to post-mortem changes. The change does not take place in living blood; for the intravenous injection of sodium chlorate in rabbits was followed by the appearance of albumen and sugar in the urine, but, in very acute cases, no methæmoglobin, which, in these animals, passes very easily from the blood to the urine. In protracted cases, however, an active hyperæmia of the kidney is set up, and the blood corpuscles which pass into the urine are the source of the methæmoglobin which has been found in the urine in many cases of chlorate-poisoning.

The poisonous action of potassium chlorate is due, according to Jacobi, to paralysis of the heart, and this is attributed, by Leichtenstern, to the action of the metal. It does not differ, according to Stokvis, from the action of potassium chloride or sulphate in corresponding doses. According to these authorities, symptoms of poisoning take place only when the dose is large, or the solution concentrated, or when a considerable quantity has been taken into an empty stomach in the form of small doses, frequently repeated. Sodium chlorate acts, according to Van Gorcom, precisely like sodium chloride, whether administered by the mouth, or by the intravenous injection of concentrated solutions.—*William B. Hills, M. D., in Boston Medical and Surgical Journal.*

